# Octreotide Use in a Patient with MEN-1 Syndrome and Multifocal Pancreatic Neuroendocrine Tumors: A Case Report and Review of the Literature

**DOI:** 10.1155/2019/9462942

**Published:** 2019-04-30

**Authors:** Matthew H. Kemm, Cory D. Manly, Thanh D. Hoang, Vinh Q. Mai, Mohamed K. M. Shakir

**Affiliations:** Division of Endocrinology, Department of Medicine, Walter Reed National Military Medical Center, Bethesda, USA

## Abstract

We report a patient with multiple endocrine neoplasia type 1 with pancreatic polypeptide (PP) secreting subcentimeter pancreatic neuroendocrine tumors (pNETs) treated with octreotide and review the current literature that pertains to the management of these patients. Clinical data, laboratory results, and imaging were reviewed. A literature search was performed in PUBMED using combinations of the terms “multiple endocrine neoplasia type 1,” “somatostatin,” octreotide,” “pancreatic polypeptide,” and “pancreatic tumor.” Relevant references were selected and reviewed. A 43-year-old male with a history of MEN1 and multiple subcentimeter neuroendocrine tumors with elevation of PP was treated with octreotide therapy leading to a reduction and normalization of PP levels. The patient tolerated octreotide therapy but self-discontinued octreotide after 24 months with a rise in PP levels off therapy. Tumors remained stable in size through 40 months of imaging follow-up. In patients with MEN1 and subcentimeter pNETs, octreotide therapy is well tolerated and can lead to a significant drop in PP levels with no change in lesion size. There is insufficient data to suggest long-term benefit with octreotide therapy but it may be considered versus standard conservative management.

## 1. Introduction

Multiple endocrine neoplasia type 1 (MEN1) is a rare, autosomal dominant syndrome causing a predisposition to tumors of the parathyroid glands, anterior pituitary, and enteropancreatic endocrine cells with an estimated prevalence of 2 to 20 per 100,000 individuals [1]. The malignant potential of pancreatic neuroendocrine tumors (pNETs) is now the primary life-threatening manifestation of MEN1 [2]. Functioning enteropancreatic endocrine tumors such as gastrinomas, insulinomas, or the rarely seen VIPomas or glucagonomas may be readily diagnosed based on clinical symptomatology alone [1]. However, nonfunctional tumors may constitute the bulk of these enteropancreatic tumors with studies suggesting a prevalence of 30-80% and with current screening protocols being likely discovered very early in the disease process [3, 4]. These asymptomatic, enteropancreatic tumors may be malignant and capable of causing liver metastases with evidence to suggest a worsened prognosis compared to functional tumors.

Current recommendations for screening for enteropancreatic neuroendocrine tumors include both biochemical evaluation and radiological screening [1]. Cost effective imaging may include magnetic resonance imaging (MRI), computed tomography (CT), or endoscopic ultrasound. It is recommended that annual biochemical evaluation includes measurement of serum gastrin, glucagon, vasoactive intestinal polypeptide, pancreatic polypeptide, chromogranin A, and insulin levels in addition to a fasting blood glucose. There are few studies evaluating the utility of these peptides in the setting of MEN1 patients. Pancreatic polypeptide (PP), a 36-amino-acid peptide produced and secreted by PP cells of the pancreas, has been shown to have a low sensitivity and specificity for pNETs in patients with MEN1, with one study reporting 36% sensitivity and 74% specificity [5–7]. However, a separate study found that, in MEN1 patients with elevated PP, a level 3.0 times the normal age-specific value was 95% sensitive and 88% specific for an imageable islet cell tumor [7].

The management of pNETs is variable due to the heterogeneity of the disease. The role of surgery for nonfunctional pancreatic tumors (NFPETs) in MEN1 is controversial with the endocrine society clinical practice guideline suggesting surgery for tumors greater than 1 cm and/or demonstrating significant growth over 6-12 months [1]. For nonresectable tumors, treatment includes somatostatin analogs (SSA), targeted radionucleotide therapy, locoregional treatments, and chemotherapy. Treatment of small pancreatic tumors in asymptomatic individuals has yet to be fully delineated in the guidelines due to lack of long-term data comparing treatment options. Observational data suggest a low potential for metastasis and no increased mortality in NFPETs under 2 cm [8]. Additionally, pNETs under 2 cm appear to grow slowly and those under 1 cm may grow even slower whereas surgical resection at any tumor size can carry substantial risks [9, 10].

Here we describe a middle-aged patient with a history of MEN1 who presented with a markedly elevated serum PP level, leading to the discovery of multiple subcentimeter pancreatic lesions found on screening CT, and with prolonged stability on SSA medical therapy.

## 2. Case Presentation

**Figure 1:**
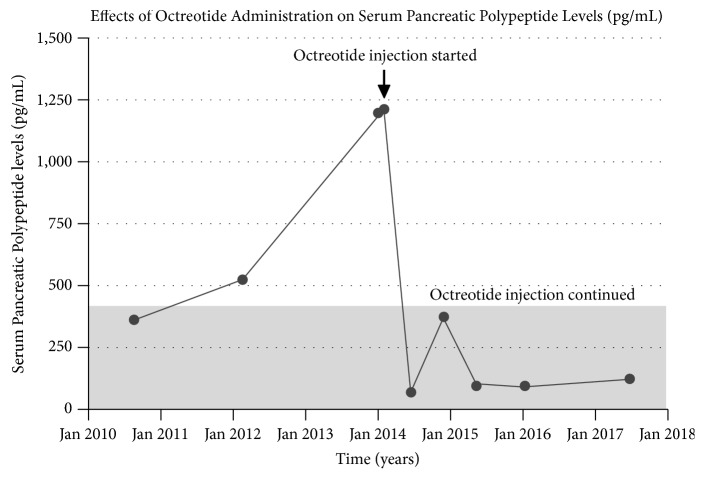
Showing serum pancreatic polypeptide (PP) levels (pg/mL) over time. Octreotide treatment was initiated in February 2014. Since initiation of octreotide treatment, the PP levels remain stable. Reference range PP (0.0-418.0 pg/mL).

**Figure 2:**
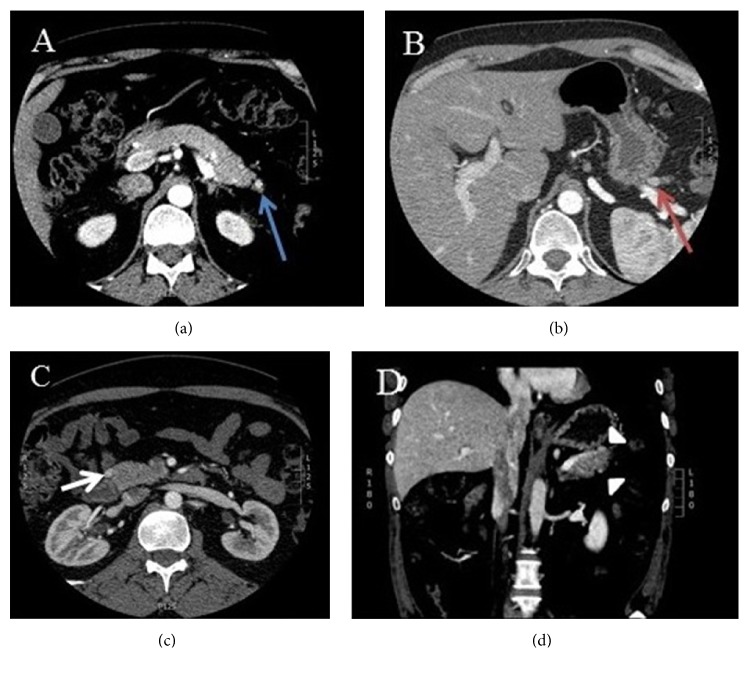
CT of the pancreas showing three hyperenhancing lesions. (a) 4 mm lesion in the inferior aspect of the pancreatic tail (blue arrow). (b) 9 mm lesion in the superior aspect of the pancreatic tail (red arrow). (c) 10 mm lesion in the pancreatic head (white arrow). (d) Coronal view revealing both pancreatic tail lesions (arrow heads).

**Figure 3:**
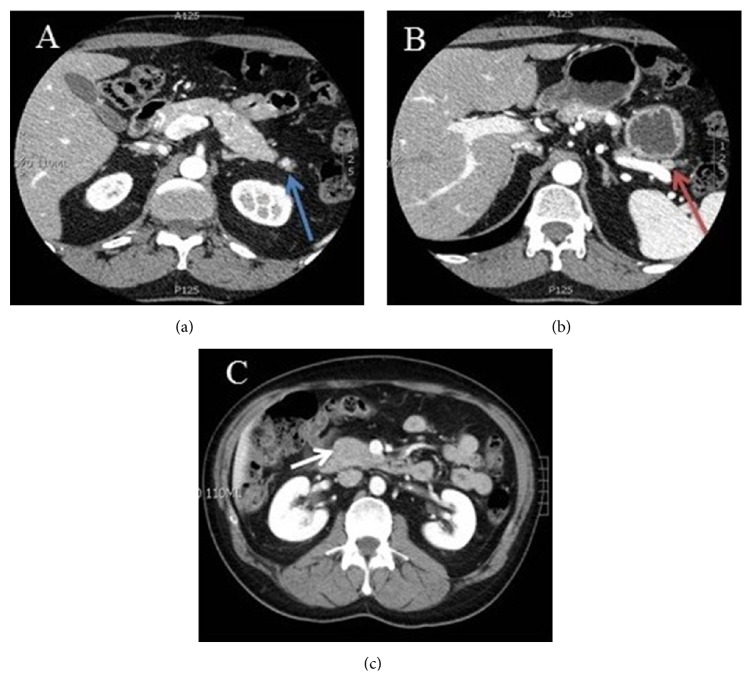
CT of the pancreas showing three hyperenhancing lesions which have remained stable 38 months after initiating octreotide. (a) Stable 4 mm tumor in the inferior aspect of the pancreatic tail (blue arrow). (b) Stable lesion in the superior aspect of the pancreatic tail (red arrow). (c) Stable lesion in the pancreatic head.

**Figure 4:**
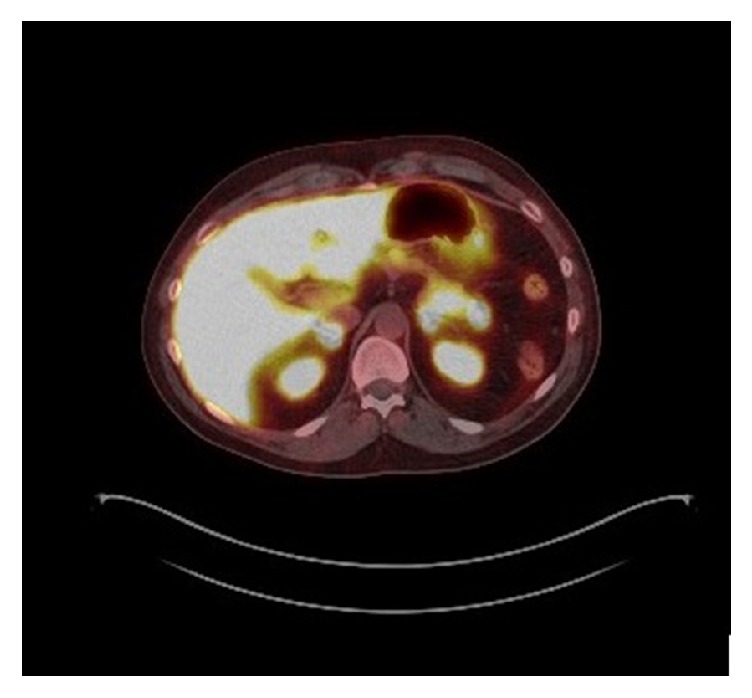
Gallium-68 DOTATATE PET/CT scan showing stability of the pancreatic tumors over time.

A 43-year-old male reported to endocrine clinic for evaluation of pancreatic lesions. His past history was relevant for total parathyroidectomy for parathyroid hyperplasia, with forearm autograph implantation at the age of 16 years. The patient has a strong family history of pancreatic and parathyroid disorders in his paternal grandfather, two paternal aunts, a paternal uncle, his father, and sister. Additionally, menin gene mutation was confirmed in his father and paternal uncle. The patient underwent a genetic testing at the age of 40 years and this confirmed menin gene mutation. Patient had no symptoms to suggest hypoglycemia, peptic ulcer disease, diarrhea, or other symptoms of endocrine disorders. Review of systems was unremarkable. On physical examination, the vital signs were normal and examination of the heart, lungs, and abdomen was also normal. He has no clinical features of hypogonadism or Cushing syndrome. The patient is married without children and does not smoke or drink alcohol. Additional blood test showed normal serum calcium, testosterone levels, FSH, LH, prolactin, and IGF-I. Additionally evaluation for Cushing syndrome was also negative. Serum gastrin, chromogranin A, vasoactive intestinal polypeptide levels, and 24-hour urine 5-HIAA levels were also normal, and a 48-hour fast did not confirm hypoglycemia. However, fasting serum pancreatic polypeptide level was elevated (520 pg/mL, reference 0-418). A CT scan of the abdomen showed multiple subcentimeter lesions. The patient refused any treatment initially; 11 months later the serum pancreatic polypeptide levels rose to 1198 pg/mL (range 912-1588), and 5 weeks later the value was 1215 pg/mL (Figure 1). At this time a repeat CT scan showed 3 hyperenhancing lesions in the head and tail of the pancreas measuring 10, 9, and 4 mm (Figure 2). These lesions correlated with 111–Indium Octreotide scan (Figure 3). Since the patient refused any surgical treatment, he was offered octreotide treatment. Initially the patient was treated with the short-acting octreotide 100 *μ*g 3 times daily, and this was later transitioned to a long-acting octreotide 20 mg monthly. Following the octreotide treatment, the serum pancreatic polypeptide levels normalized to 62 pg/mL in approximately 6 weeks (Figure 1). A follow-up CT scan at 9 and 33 months later confirmed stability of the pancreatic lesions without evidence of metastasis. Additionally, the serum pancreatic polypeptide levels remained within normal limits (91.6 pg/mL) (Figure 1). The patient continued to feel well and has tolerated octreotide without any adverse effects over the entire period of treatment. A most recent gallium-68 DOTATATE PET/CT scan (Figure 4) performed 3 years later, while the patient was on octreotide therapy, further confirmed the stability of the pancreatic lesions. Additionally, serum pancreatic polypeptide remained normal along with normal levels of somatostatin, glucagon, gastrin, vasoactive intestinal polypeptide, and chromogranin A during the entire course of octreotide treatment.

## 3. Discussion

Use of somatostatin analogs in the treatment of pNETs is based on investigations showing that the natural 14-amino-acid peptide binds to G-protein-coupled somatostatin receptors (SSTRs) that are expressed on most NETs with SSTR-2 expressed in approximately 80% of pNETs [11, 12]. Octreotide and lanreotide bind with a high affinity to SSTR-2 [13, 14]. Binding to these receptors can lead to antisecretory and antiproliferative effects. It has been shown that significant improvement in patients with gastrinoma, VIPoma, and glucagonoma can occur following somatostatin analog treatment [15]. In the phase III CLARINET trial of 204 patients, lanreotide 120 mg, in patients with advanced, well or moderately differentiated nonfunctional GI and pancreatic NETs with SSTR avid disease, leading to significantly prolonged median progression-free survival (>24 mo.) vs. placebo (18 mo.) [16]. Given the relative good safety profile of somatostatin analog therapy, the results of the CLARINET trial significantly question the utility of observation in patients with locally, advanced, unresectable, or metastatic NETs and support the inclusion of SSA treatment in early stage NET management. National Comprehensive Cancer Network (NCCN) guidelines now state that treatment can be considered for these patients with sporadic NFPETs [17]. Whether this would be the case in an NFPET in MEN1 is unknown. Results from the Dutch MEN1 and the French Endocrine Tumor study groups have recently shown that surgical treatment of NFPET nodules <2cm versus observation resulted in no significant increase in survival over time but did cause a significant number of postoperative complications [8, 18]. If surgery is no longer a reasonable treatment option for this group of patients and observation is not a reasonable option for a patient, SSA therapy may be an appropriate alternative. The currently available data suggest that there may be a benefit in these small, <2 cm, nonfunctional nodules. In a single study in twenty MEN1 patients on SSA therapy over 12-75 months of follow-up with tumors <2 cm, stable disease was found in 80% of patients, tumor shrinkage in 10%, and hormonal suppression in all 6 patients with elevated CgA or gastrin levels [19]. Cioppi et al. (LARO-MEN1 study) investigated whether early treatment with long-acting somatostatin analog could act as a preventive approach in relatively small MEN1-related gastroenteropancreatic neuroendocrine tumors. In 8 patients with small (<2 cm) neuroendocrine tumors and abnormal laboratory values of at least one of the gastroenteropancreatic hormones, administration of octreotide acetate slow-release formulation (LAR) (10 mg intramuscularly every 28 days) was effective in decreasing gastroenteropancreatic hormone levels and stabilizing tumor size. Furthermore the treatment was safe in the majority of patients up to six years of treatment [20]. Additional data in this select population is likely many years away but a phase 3 trial in Europe comparing SSA therapy vs. no treatment in MEN1 patients with NFPET will be opening soon for enrollment [21].

Additionally, there is insufficient evidence to clarify whether a decline in PP level correlates with a reduction in tumor burden. We would argue that the significant decline in PP level, a nonspecific tumor marker, in addition to the lack of tumor growth and stable follow-up PP level is indicative of a treatment effect as has been seen with other hormonally active NETs treated with SSA therapy. We speculate that if the level of PP continues to rise off therapy, the patient would experience further nodular growth, which could have adverse health consequences. Typical management of subcentimeter NFPETs has typically involved serial imaging and biochemical testing. SSA therapy in addition to serial testing represents a reasonable treatment option that is well tolerated in this patient population although its high-cost may hinder its utilization.

Furthermore, basal and meal-stimulated pancreatic polypeptide has been shown to be useful for early detection of pancreatic involvement in 75% of patients with MEN-1. Our patient's basal fasting pancreatic polypeptide levels were not only elevated but actually doubled at one-year follow-up. The stability of the pancreatic tumors along with the normalization of serum PP level confirms the effectiveness of SSA therapy in this patient. It may be stated that the pancreatic lesions would have remained stable without octreotide treatment; however, octreotide was effective in our patient for several reasons: (1) the serum PP level doubled over the 11-month observation period when he did not receive octreotide treatment; (2) there was an increase in the size of pancreatic tumors during this observation period; and (3) a gallium-68 DOTATATE scan showed stability of tumor size over the course of treatment. Therefore, these findings support the effectiveness of octreotide treatment. As the benefits of pancreatic surgery for small lesions (<2cm) are not clear, the morbidity, mortality, and long-term complications of surgery must be considered. Our patient was thus offered medical therapy with long-acting octreotide, which was recently shown to provide 90% objective tumor response and stability in MEN-1 patients with early nonfunctional pancreatic NET [19]. Discontinuation of octreotide and reassessment of serum pancreatic polypeptide level and tumor growth would have confirmed the effectiveness of octreotide therapy; however, we felt it may be unethical to discontinue octreotide therapy.

## 4. Conclusion

The malignant potential of pNETs is the primary life-threatening manifestation of MEN1 but treatment for NFPET <1 cm has not been clearly defined by the guidelines. We describe the case of a middle-aged patient with a history of MEN1 who presented with elevated serum PP levels and multiple subcentimeter pancreatic lesions found on imaging who was treated with SSA therapy for 24 months leading to a marked reduction in PP level during treatment with stability in lesion size after 40 months of follow-up. SSA therapy represents a reasonable and potential alternative to watchful waiting in this patient population but further research is necessary to better define long-term outcomes.

## Abbreviations

CgA: Chromogranin A
CT: Computed tomography
MEN1: Multiple endocrine neoplasia type 1
NFPET: Nonfunctional pancreatic tumor
pNET: Pancreatic neuroendocrine tumor
PP: Pancreatic polypeptide
VIP: Vasoactive intestinal peptide
PET: Positron emission tomography.
